# Special Care in Lichen Planus Patients Undergoing LASIK: A Review Article

**Published:** 2019

**Authors:** Majid Moshirfar, Harry Y. Liu, David B. Rosen, Madeline B. Heiland, Masoud Mirghorbani, Yasmyne C. Ronquillo, Phillip C. Hoopes

**Affiliations:** 1 John A. Moran Eye Center, Department of Ophthalmology and Visual Sciences, School of Medicine, University of Utah Salt Lake City, UT, USA; 2 Utah Lions Eye Bank, Murray, UT, USA; 3 Hoopes Durrie Rivera Research Center, Hoopes Vision, Draper, UT, USA; 4 McGovern Medical School, Health Science Center, University of Texas, Houston, TX, USA; 5 College of Medicine-Phoenix, University of Arizona, Phoenix, AZ, USA; 6 Eye Research Center, Farabi Eye Hospital, Tehran University of Medical Sciences, Tehran, Iran

**Keywords:** Lichen Planus, Conjunctivitis, Lichenoid Eruptions, Keratomileusis, LASIK

## Abstract

Laser-Assisted in Situ Keratomileusis (LASIK) is a common surgery for the correction of refractive errors. The majority of patients who undergo this procedure often have excellent results. However, uncontrolled autoimmune disorders and dry eye have both been listed as contraindications to this surgery. Lichen planus (LP) is an autoimmune, inflammatory disorder that characteristically affects mucocutaneous membranes. The etiology is unknown, but it most commonly affects middle-aged adults and presents with bilateral, purple papules. Clinical presentation is used to diagnose the condition, and a punch biopsy is confirmatory. LP may present with multiple different symptoms depending on the type, with ocular manifestations being rare. Multiple viruses and autoimmune conditions have been associated with the disorder, and physicians should take care when gathering a full history of the patient. Exacerbation of symptoms may happen if mood disorders such as depression and anxiety are not well controlled. There are several additional factors physicians must carefully consider before recommending LASIK to patients with LP. These include lichenoid reactions, current medications, and past or present ocular lesions. LASIK may be carefully considered in patients with well-controlled LP in the absence of ocular manifestations. Patients with ocular LP are not candidates for LASIK.

## INTRODUCTION

Laser-Assisted in Situ Keratomileusis (LASIK) is a common surgical option for the correction of refractive errors. In appropriately selected patients, excellent uncorrected distance vision may be achieved in more than 90% of cases [1]. In general, patients with uncontrolled autoimmune disorders do not undergo the procedure [2]. One such disorder is Lichen planus (LP), which can present with ocular manifestations. This raises concerns about healing after LASIK due to abnormalities in the ocular surface such as dry eye, blepharitis, and conjunctival cicatricial changes [3]. These complications not only interfere with suction and flap creation during the procedure but may also result in improper postoperative wound healing. Although uncommon, ophthalmologists will likely encounter LP patients in the clinical setting. 

We aim to highlight considerations given to patients with LP seeking corneal refractive surgery.

## METHODS

To find information on LP, a literature search was performed using the following sources: PubMed, Google Scholar, Embase, and Scopus with the keywords “Lichen planus,” “ocular manifestations of lichen planus,” “cornea lichen planus,” and “conjunctiva lichen planus.” Reference lists of these articles were used to find additional articles. There were no language restrictions. Publications were drawn between the dates of 1990-2019.

Lichen Planus and LASIK

**Figure 1:**
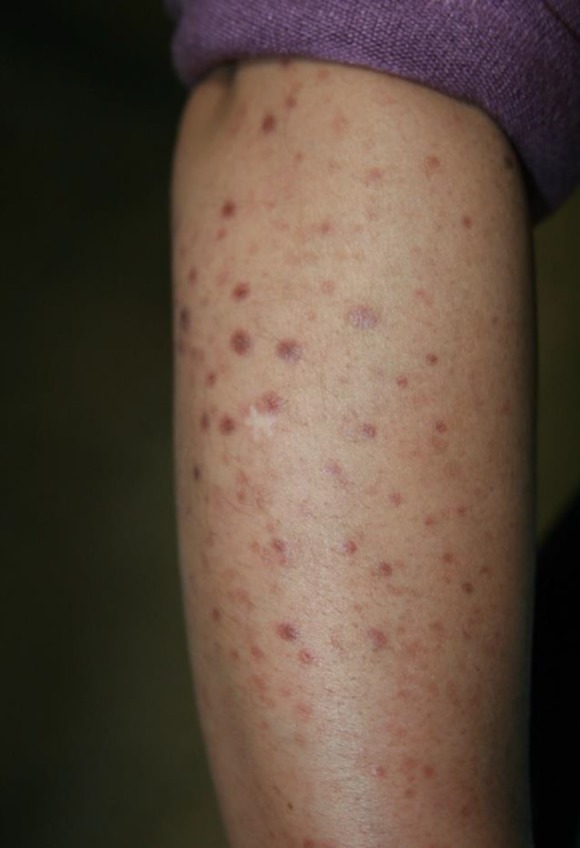
Pruritic and Purple Papules on the Right Forearm of a 43 Years Old Woman with a Diagnosis of Cutaneous Lichen Planus (LP) (Courtesy of Dr. Mirghorbani)

LP is a chronic, inflammatory autoimmune disorder affecting mucocutaneous membranes such as the skin, oral cavity, and vagina. Cutaneous LP, the most common type, is classically characterized by pruritic, purple, bilateral papules (Fig. 1) [4, 5]. The disorder is uncommon, presenting in roughly 1% of the population [5]. Middle-aged adults are most commonly affected by LP, with the disorder showing a slight female predominance [6].

The etiology of LP is unknown, but mechanisms have been hypothesized [5]. LP involves a T-cell mediated response that recruits dendritic cells into layers of the epithelium [7]. This occurs as a result of inflammation caused by altered self-antigens [7]. It is a clinical diagnosis confirmed by punch biopsy [4, 5]. Histopathology reveals lymphocyte infiltration and IgM deposition in the dermal-epidermal junction, which helps differentiate LP from other autoimmune disorders [4, 5, 8]. 

Hepatitis C virus (HCV), Human Papillomavirus (HPV), and Epstein-Barr virus (EBV) are seen more frequently in patients with LP [4, 9]. HCV occurs five times more often in patients with LP [4]. There is a significantly increased prevalence of HPV and EBV in LP patients [4, 9]. Human Papillomavirus can cause malignant transformation of LP lesions, but it is inconclusive whether EBV may do the same [9, 10]. Stress, anxiety, and mood disorders have shown to increase the risk of exacerbations, with stress specifically known to induce lesions in asymptomatic individuals [4]. Because of this, mood disorders should be well controlled before and after LASIK. A few reports have also noted LP to occur in conjunction with other autoimmune disorders including Sjogren’s Syndrome and Systemic Lupus Erythematosus, but no relationship has been discovered [4, 8, 11]. 

**Figure 2:**
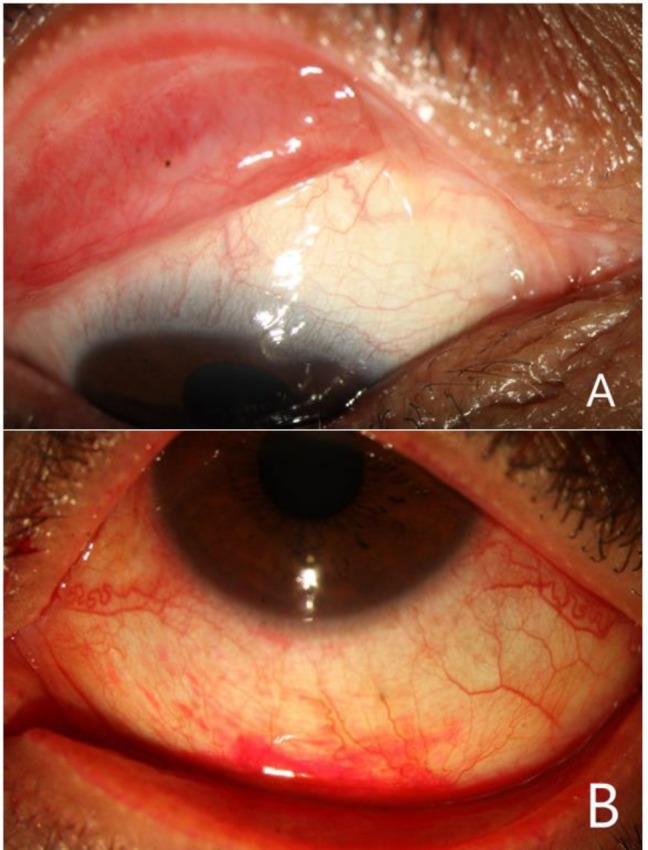
Ocular Involvement was detected in the Same Patient Mentioned in Figure 1. A: Obvious Fornix Shortening and Medial Symblepharon of the Upper Eyelid. B: Inferior Bulbar and Forniceal Conjunctival Staining with Rose Bengal Indicating Mucosal Disruption of these Areas (Photos Courtesy of Dr. Mirghorbani)

Development of ocular symptoms is extremely rare, as in the last twenty years only 40 cases of ocular involvement have been documented [3]. Ocular manifestations of LP can include cicatrizing conjunctivitis, blepharitis, and lacrimal duct obstruction [12-14]. Conjunctivitis ranges from inflammation to scarring of the conjunctiva, which can lead to subepithelial fibrosis and dry eye (Fig. 2) [13, 15]. Obstruction of the lacrimal ducts can lead to epiphora and increased risk of infection [3]. Through damage to the Meibomian glands, blepharitis can cause decreased tear production and worsening dry eye symptoms [13]. Many of the ocular symptoms have been seen together [3]. Corneal manifestations of LP independent from conjunctivitis are incredibly rare and have only been documented once [3, 15]. This case presented with pigmented spots on the cornea [3, 15]. One diagnostic challenge that physicians face is determining whether a patient presents with true LP or a lichenoid reaction. Lichenoid reactions are lesions caused by medications, which may persist weeks to months after cessation of the offending drug [4]. Common causes of lichenoid reactions include antimicrobials, insulin, sulfonylureas, anti-inflammatories, antimalarials, antipsychotics, chemotherapeutics, and cardiovascular drugs [4]. Contact lichenoid reactions may occur from natural flavorings and metals used in dental procedures [4].

First-line treatments for cutaneous and oral LP are acitretin (a retinoid) and topical corticosteroids respectively [16]. During clinical trials, 10%-25% of patients on acitretin developed dry eyes [17]. In addition, there are rare reports of corneal ulceration and keratitis related to the use of acitretin [18]. Because of this, we recommend that patients undergo evaluation for adequate tear production via Schirmer tear test and tear quality via tear film breakup time [19]. If these values are within normal limits, LASIK may be considered. For widespread cases of LP, high-dose systemic corticosteroids of 0.5-1.0 mg/kg/day are the treatment of choice [20]. Treatment regimens for these cases last for at least four weeks before tapering off to a lower maintenance dose [5, 20]. However, corticosteroid use for at least 30 days preoperatively at doses of 40 mg/day has shown to increase wound complication rates 2-5 times [21]. We do not recommend LASIK if patients are taking corticosteroids more than 40 mg/day.

Recently, biologics, including Rituximab, have been shown to be effective treatments for LP [22, 23]. However, Rituximab has been associated with Herpes Simplex Virus keratitis [24]. The combination of topical Cyclosporine A (CsA) and a corticosteroid has shown to be effective in treating conjunctivitis and corneal lesions in LP [3, 25, 26]. However, this medication regimen only seems to delay progression; after cessation of treatment, symptoms worsen [3]. While CsA is an immunosuppressant, it does not appear to cause an increased risk for opportunistic infections [27]. There are reports of ocular findings related to CsA, but these appear to require high doses of systemic therapy [28]. While treatment with biologics is not an absolute contraindication to LASIK, we recommend close post-op monitoring with a low threshold to treat. 

Due to the chronic and irreversible nature of the changes caused by ocular LP, it is generally not recommended that patients with ocular symptoms undergo LASIK or any other corneal refractive surgeries [3]. The corneal flap created by LASIK can increase symptoms of dry eye, which combined with LP-related ocular manifestations can lead to severe dry eye, corneal melt, and potentially corneal perforation. LP can be exacerbated by a multitude of factors ranging from anxiety to trauma [4, 26]. Patients with ocular LP should understand that any visual improvement made with LASIK may reverse during the next exacerbation of LP.

Physicians should be aware of a few pre-operative and postoperative considerations, including current or past ocular symptoms, coexisting autoimmune conditions, medications that can cause a lichenoid reaction, and LP treatment regimens. A multi-disciplinary approach, including input from dermatology and rheumatology, is recommended. A complete ocular examination paying specific attention for small symblepharon, fornix shortening, adequate tear production, and probing of the canaliculi and the nasolacrimal duct is strongly suggested as these may predict the course of the disease after any ocular procedure (Fig. 2A). Conjunctival staining with rose bengal and lissamine green are important tests to evaluate ocular surface integrity (Fig. 2B) as they show mucosal disruption. Emphasis should be placed on frequent lubrication post-operatively, and any epithelial insults should be managed aggressively and immediately. Informed consent must be obtained with an emphasis on risk for severe dry eye, keratitis, corneal melt, and corneal perforation. However, ocular findings are exceedingly rare, and LASIK may be a reasonable option in patients with well-controlled LP in the absence of ocular involvement. Table 1 is a summary of pre- and Post-LASIK considerations in LP patients.

**Table 1:** Pre- and Post-LASIK Considerations For Physicians Assessing Lichen Planus Patients For Laser-Assisted in Situ Keratomileusis (LASIK).

Special care in Lichen planus patients undergoing LASIK
Pre-operative assessments
**Medications causing a lichenoid reaction**
**Ask patient about history of Epstein-Barr virus, Human Papillomavirus, Hepatitis C virus infections**
**Consultation with dermatologist/rheumatologist**
**Psychiatric consultation if patients have comorbid anxiety/mood disorder**
**Detailed history concerning past or present use of biologics/corticosteroids/retinoids such as acitretin**
**Slit-lamp examination with staining looking for fornix shortening, small symblepharon, mucosal disruption**
**Probing of canaliculi and nasolacrimal ducts**
**Comprehensive review of symptoms investigating past or recent exacerbations of Lichen planus**
**Coexisting autoimmune disorders (e.g. Systemic Lupus Erythematosus, Sjogren’s Syndrome)**
Post-operative considerations
**Careful monitoring and control of coexisting autoimmune disorders**
**Control anxiety/any mood disorders**
**Increased risk of delayed wound healing, dry eye, corneal melt, corneal perforation, and viral or bacterial keratitis**
**Aggressive management with a low threshold to treat any symptoms of dry eye**

## CONCLUSION

Like most autoimmune conditions, patients with LP must be carefully evaluated before considering them for elective refractive procedures such as LASIK. LP can present with a multitude of symptoms, requiring a multidisciplinary approach to care. When well-controlled, patients with this disorder may reasonably undergo LASIK after a comprehensive physical and history. Patients should be informed that ocular manifestations of LP can lead to permanent damage to the cornea, and that refractive surgeries such as LASIK are a high risk procedure to them.

## DISCLOSURE

Ethical issues have been completely observed by the authors. All named authors meet the International Committee of Medical Journal Editors (ICMJE) criteria for authorship of this manuscript, take responsibility for the integrity of the work as a whole, and have given final approval for the version to be published. No conflict of interest has been presented.

Funding/Support: Research to Prevent Blindness, NY, USA
